# Clinical usefulness of end‐tidal CO_2_ measured using a portable capnometer in patients with respiratory disease

**DOI:** 10.1111/crj.13577

**Published:** 2023-01-06

**Authors:** Manabu Suzuki, Shota Fujimoto, Keita Sakamoto, Kentaro Tamura, Satoru Ishii, Motoyasu Iikura, Shinyu Izumi, Yuichiro Takeda, Masayuki Hojo, Haruhito Sugiyama

**Affiliations:** ^1^ Department of Respiratory Medicine National Center for Global Health and Medicine Tokyo Japan

**Keywords:** capnometer, EtCO_2_, PaCO_2_, PvCO_2_, respiratory care, respiratory medicine

## Abstract

**Introduction:**

This study aimed to evaluate the correlation and agreement between end‐tidal CO_2_ (EtCO_2_) measured with the novel portable capnometer (CapnoEye®) and partial pressure of arterial carbon dioxide (PaCO_2_) levels in patients with respiratory diseases and to compare the efficacy of EtCO_2_ and PvCO_2_ in predicting PaCO_2_ levels.

**Methods:**

We analyzed the correlation and the agreement between EtCO_2_ and PaCO_2_ and between PvCO_2_ and PaCO_2_ using Pearson's moment correlation coefficient in patients with type 1 and type 2 respiratory failure and both groups overall.

**Results:**

A total of 100 samples were included that comprised 67 men (67%). The mean age of the subjects was 77 ± 13 years. Chronic obstructive pulmonary disease (COPD) (43%) was the most common disease. There was a high correlation between EtCO_2_ and PaCO_2_ (*r* = 0.88; *p* < 0.0001). Sixty‐six PvCO_2_ samples were obtained, and there was a high correlation between PvCO_2_ and PaCO_2_ (*r* = 0.81; *p* < 0.0001). Regarding type 2 respiratory failure, there was a high correlation between EtCO_2_ and PaCO_2_ (*r* = 0.81). The Bland–Altman analysis between PaCO_2_ and EtCO_2_ revealed a bias of 5.7 mmHg, with limits of agreement ranging from −5.1 mmHg to 16.5 mmHg. In contrast, the analysis between PaCO_2_ and PvCO_2_ revealed a bias of −6.8 mmHg, and the limits of agreement ranged from −22.13 mmHg to 8.53 mmHg.

**Conclusion:**

EtCO_2_ measured by CapnoEye® was significantly correlated to PaCO_2_ levels in patients with respiratory diseases. Moreover, CapnoEye® may be more useful for predicting hypercapnia conditions in which respiratory diseases are compared with measure PvCO_2_.

## Graphical abstract

EtCO_2_ measured by CapnoEye® was significantly correlated to PaCO_2_ levels in patients with respiratory diseases. While type 1 patients displayed a moderate correlation, type 2 patients demonstrated a high correlation between EtCO_2_ and PaCO_2_. In contrast, the correlation between PvCO_2_ and PaCO_2_ was moderate for both type 1 and type 2. CapnoEye® may be more useful for predicting hypercapnia conditions in which respiratory diseases are compared with measure PvCO_2_.

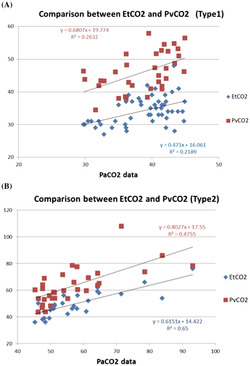

**Abbreviations**
ABG: arterial blood gas
COPD: chronic obstructive pulmonary disease
EtCO_2_
: end‐tidal carbon dioxide
ICU: intensive care unit
PaCO_2_
: partial pressure of arterial carbon dioxide
PaO_2_
: partial pressure of arterial oxygen
PvCO_2_
: partial pressure of venous carbon dioxide

## 1 INTRODUCTION

Respiratory failure is a clinical condition characterized by the failure of the respiratory system to maintain its major function, that is, gas exchange, in which the partial pressure of arterial oxygen (PaO_2_) is <60 mmHg and/or the partial pressure of arterial carbon dioxide (PaCO_2_) is >45 mmHg. Respiratory failure is classified according to blood gas abnormalities into types 1 and 2. Type 1 (hypoxemic) respiratory failure is a disorder of the respiratory system in which the PaO_2_ and PaCO_2_ are ≤60 mmHg and <45 mmHg, respectively, due to respiratory dysfunction. A PaCO_2_ > 45 mmHg is defined as type 2 (hypercapnic) respiratory failure. Hypoxemia is a common condition and can be attributed to ventilation failure.^1^, ^2^

Arterial blood gas (ABG) assessment is essential to confirm the diagnosis of respiratory failure, and frequent measurements are necessary to confirm the respiratory status during the course of treatment. An American Thoracic Society/European Respiratory Society position paper reported on ABG analysis as the preferred method for determining the need for oxygen, as it includes acid–base information.^3^, ^4^ Blood gas analysis can assess a patient's respiratory status in real time. However, it requires repeated puncture of the peripheral arteries and is an invasive and painful medical procedure.

The monitoring of respiratory condition necessitates the most non‐invasive technique in the perioperative period and the intensive care unit (ICU).^5^ This also applies to general wards and outpatient clinics. Pulse oximetry is commonly used to evaluate oxygenation levels owing to its simplicity and non‐invasive design. Nonetheless, it does not provide additional information regarding the adequacy of ventilation or precise arterial oxygenation, particularly when arterial oxygen levels are extremely high or low.

The measurement of end‐tidal carbon dioxide (EtCO_2_) is another non‐invasive method for estimating PaCO_2_. EtCO_2_ is generally measured as biological information in patients in the operating room or in the ICU with controlled ventilation. However, it is not usually measured in general wards or laboratories.^6^ A possible reason for its unpopularity in general wards is the unfeasibility to collect pure respiratory gas in non‐intubated patients, thus increasing the difficulty to accurately measure EtCO_2_.^7^

The novel portable capnometer (CapnoEye®, NISSEI Ltd., Japan) can non‐invasively measure EtCO_2_ not only at the patient's bedside or outpatient clinic but also during medical home‐visits. The measurements can be easily and rapidly conducted using six spontaneous breaths. However, it is unknown whether EtCO_2_ measurements using this novel device in patients with refractory failure and spontaneous breathing can be considered an alternative to PaCO_2_ measurements that use arterial blood gas analysis in clinical settings.

We aimed to evaluate the accuracy and efficacy of the novel portable capnometer for predicting PaCO_2_ levels. CapnoEye® was used to measure EtCO_2_ and PaCO_2_ in patients with type 1 and 2 respiratory failure. We aimed to calculate and evaluate the correlation coefficients and their measurement accuracies. We also intended to compare differences between venous and arterial carbon dioxide levels only in patients who simultaneously underwent venous blood gas measurements. Furthermore, we also aimed to compare the efficacy of EtCO_2_ and PvCO_2_ in predicting PaCO_2_ levels.

## 2 MATERIALS AND METHODS

**FIGURE 1:**
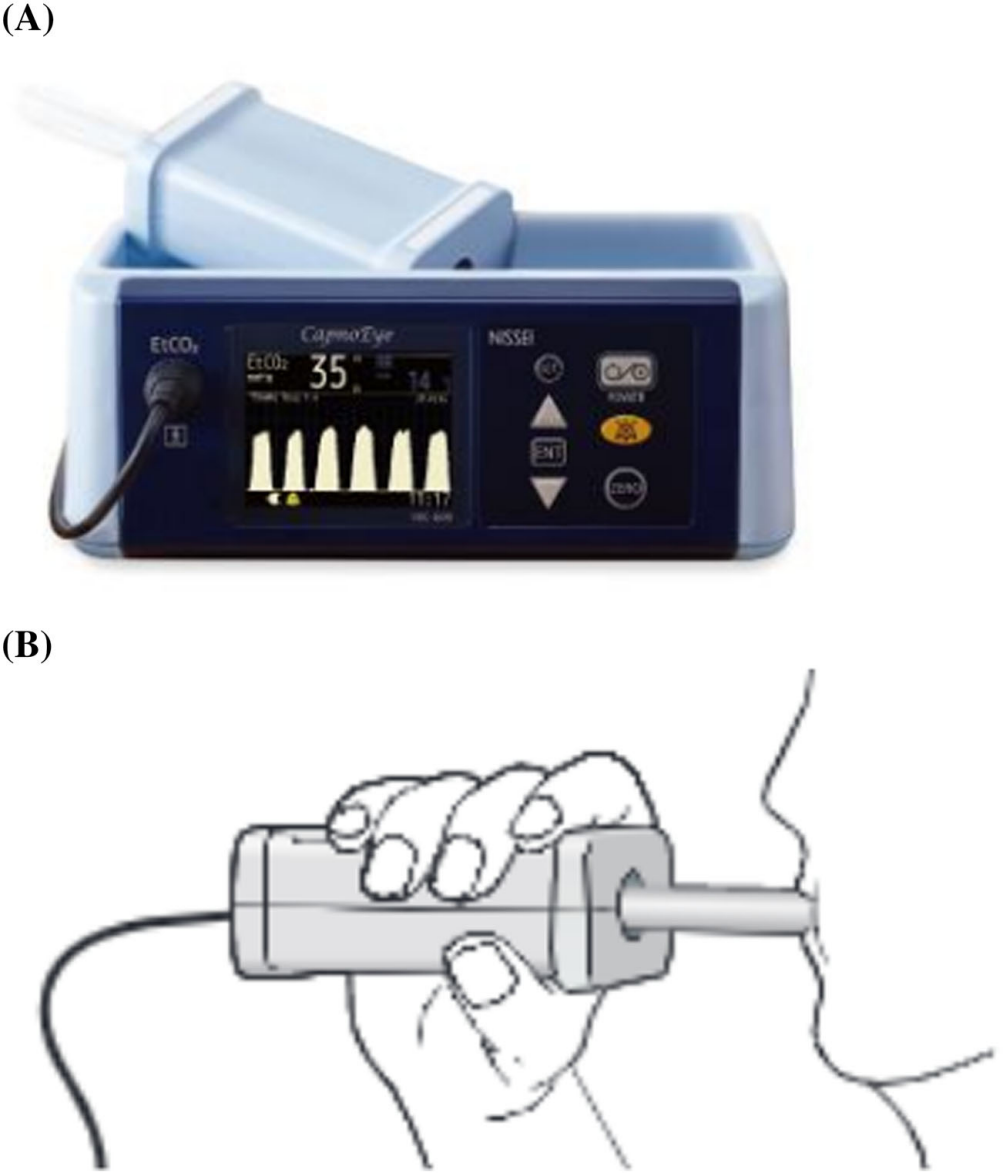
Equipment used in this study. (A) CapnoEye®. (B) EtCO_2_ measurement schema by CapnoEye®. EtCO_2_, end‐tidal carbon dioxide

We retrospectively collected clinical data of patients with respiratory diseases whose EtCO_2_ was measured from February 2017 to December 2019. Simultaneously, we collected arterial blood gas sampling to measure the PaCO_2_. Moreover, we only compared the venous carbon dioxide (PvCO_2_) and PaCO_2_ values in patients who had underwent the sampling concurrently. These samples were collected under similar conditions at a particular time. EtCO_2_ was measured using the CapnoEye® (Figure 1). EtCO_2_ measurements were performed using a mouthpiece to collect the exhaled sample, similar to the conventional capnometer. The subjects held the mouthpiece in their mouth and breathed quietly six times. The sample was measured directly as the main stream. EtCO_2_ measurement principles were based on a nondispersive infrared absorption method, which utilizes the ability to absorb infrared light of a wavelength corresponding to the concentration of carbon dioxide contained in the expired gas. Infrared light is irradiated toward and absorbed by the respiratory gas, and the amount of remaining light is detected by a light receiver through 2 types of optical filters.

### 2.1 Statistical analyses

We collected a total of 100 samples of EtCO_2_ and PaCO_2_ (51 patients with type 1 and 49 patients with type 2 respiratory failure). The measured data were compared overall and between the two groups. We collected 66 samples of PvCO_2_ (34 patients with type 1 and 32 patients with type 2 respiratory failure). The PvCO_2_ and EtCO_2_ data were also compared overall and between the groups.

We analyzed the correlation between EtCO_2_ and PaCO_2_ and between PvCO_2_ and PaCO_2_ using Pearson's moment correlation coefficient in patients with type 1 and type 2 respiratory failure and both groups overall. We also analyzed the agreement between EtCO_2_ and PaCO_2_ and between PvCO_2_ and PaCO_2_ using Bland–Altman analysis. Moreover, we calculated the estimated bias (i.e., the mean difference between the two methods) and precision (i.e., 1 SD of the differences) in subjects with type 1 and type 2 respiratory failure and both groups overall. Data are presented as the mean (standard deviation). Descriptive data are presented as the median, frequency, and percentage. Statistical analyses were performed using JMP 14 (SAS Institute, Cary, NC, USA).

### 2.2 Ethical consideration

This study was conducted in accordance with the tenets of the Declaration of Helsinki, and the protocol was approved by the Ethics Committee of Clinical Investigation for the National Center for Global Health and Medicine (approval number: NCGM‐G‐002135–00). All subjects provided informed consent for their inclusion in this study.

## 3 RESULTS

Table 1 summarizes the baseline characteristics of the study patients. The patients comprised 67 men (67%). Their mean age, body mass index, PaCO_2_, EtCO_2_, and PvCO_2_ were 74 ± 13 years, 22.0 ± 4.6, 47.2 ± 11.8 mmHg, 41.5 ± 10.1 mmHg, and 53.4 ± 13.2 mmHg, respectively. Table 2 enlists the patients with underlying diseases. Of these patients, 43 (43%) had chronic obstructive pulmonary disease (COPD), 18 (18%) had interstitial pneumonia, and 12 (12%) had old tuberculosis.

**TABLE 1:** Subject characteristics (*n* = 100 samples [80 patients])

	All samples (*n* = 100)	Type 1 (*n* = 51)	Type 2 (*n* = 49)
Gender, Male	67 (67%)	41 (80%)	21 (43%)
Age (years)	74.0 (12.9)	73.8 (12.1)	71.6 (14.8)
BMI	21.9 (4.6)	23.0 (4.3)	20.5 (4.9)
PaCO_2_ (mmHg)	47.2 (11.8)	38.6 (4.58)	55.9 (10.6)
EtCO_2_ (mmHg)	41.5 (10.1)	33.4 (4.58)	48.9 (9.0)
PaCO_2_‐EtCO_2_	5.7 (5.5)	4.4 (4.4)	7.0 (6.2)
PvCO_2_ (mmHg)	53.4 (13.2)^a^	46.1 (5.9)^b^	63.0 (13.4)^c^
PaCO_2_‐PvCO_2_	−6.8 (7.8)^a^	−7.4 (5.3)^b^	−6.4 (10.0)^c^

*Note*: Data are presented as mean (standard deviation) or *n* (%).

Abbreviations: EtCO_2_, end‐tidal carbon dioxide; PaCO_2_, partial pressure of arterial carbon dioxide; PvCO_2_, partial pressure of venous carbon dioxide; Type 1, patients with type 1 respiratory failure; Type 2, patients with type 2 respiratory failure.

^a^ *n* = 66.

^b^ *n* = 34.

^c^ *n* = 32.

**TABLE 2:** Underlying disease (*n* = 100 samples [80 patients])

	All patients (*n* = 100)	Type 1 (*n* = 51)	Type 2 (*n* = 49)
COPD	43 (43%)	25 (49%)	18 (37%)
COPD + Asthma	4 (4%)	2 (4%)	2 (4%)
Asthma	8 (8%)	3 (6%)	5 (10%)
Interstitial pneumonia	1 8 (18%)	13 (25%)	5 (10%)
Previous tuberculosis	12 (12%)	3 (6%)	9 (18%)
Thoracoplasty	6 (6%)	0 (0%)	6 (12%)
Bronchiectasis/chronic bronchitis	11 (11%)	4 (8%)	7 (14%)
Chronic lower respiratory tract infection	6 (6%)	1 (2%)	5 (10%)
Lung cancer/malignancy	6 (6%)	5 (10%)	1 (2%)
Pneumonia	10 (10%)	8 (16%)	2 (4%)
Pneumothorax	4 (4%)	1 (2%)	3 (6%)
Non‐tuberculous mycobacterium	2 (2%)	1 (2%)	1 (2%)
Chronic pulmonary aspergillosis	1 (1%)	0 (0%)	1(2%)
Pulmonary thromboembolism	2 (2%)	2 (4%)	0 (0%)
Secondary pulmonary hypertension	3 (3%)	1(2%)	2 (%)
Chronic heart failure	11 (11%)	5 (10%)	6 (12%)
Neuromuscular disease	4 (4%)	0 (0%)	4 (8%)

*Note*: When several patients had multiple diseases, we counted the number of duplicate cases.

Abbreviation: COPD, chronic obstructive pulmonary disease.

**FIGURE 2:**
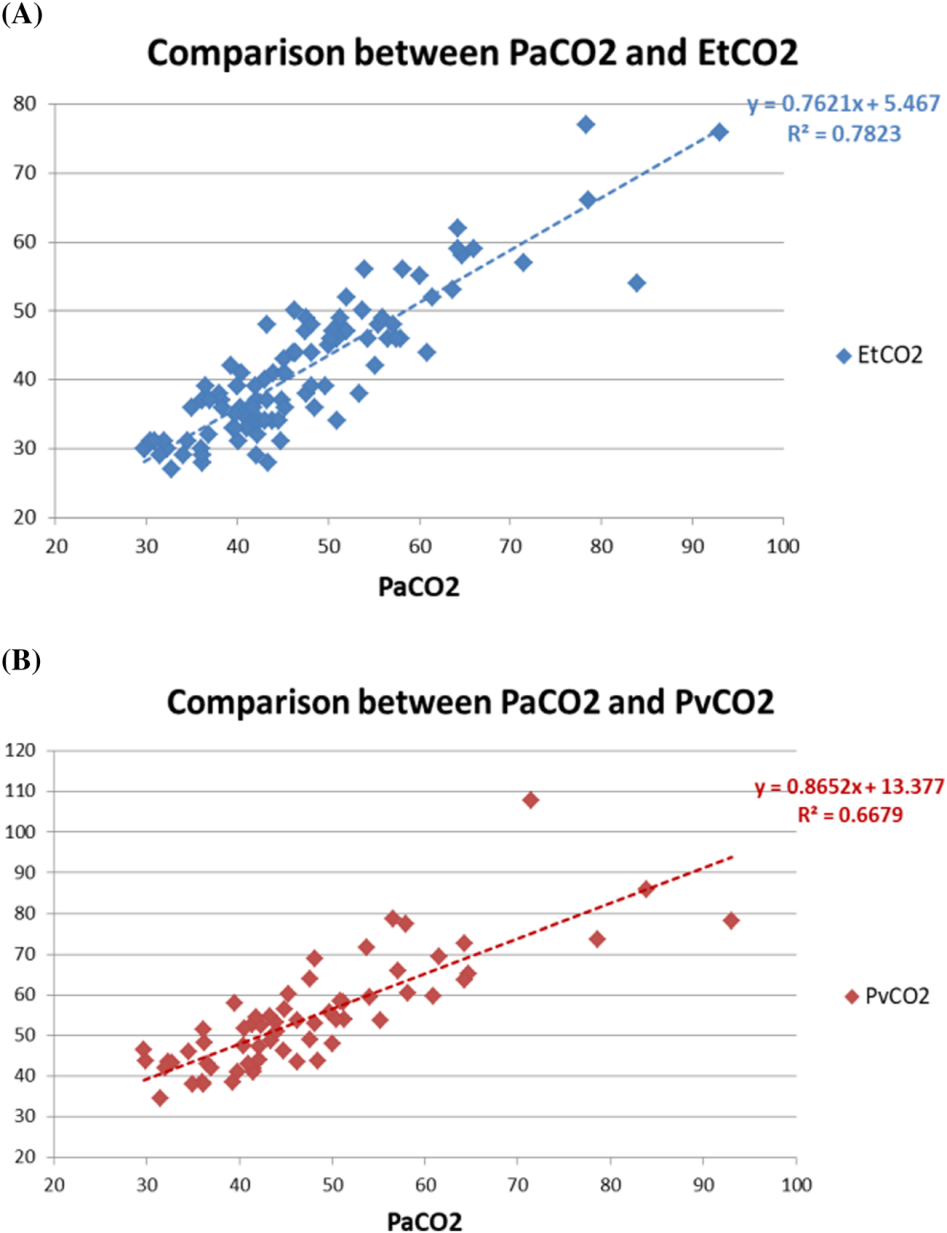
Correlation analysis plot between EtCO_2_ and PaCO_2_ and between PvCO_2_ and PaCO_2_. (A) Correlation between EtCO_2_ and PaCO_2_ in all patients (Type 1: *n* = 51, Type 2: *n* = 49). The correlation coefficient is 0.883, displaying a high positive correlation. (B) Correlation between PvCO_2_ and PaCO_2_ in all patients (Type 1: *n* = 34, Type 2: *n* = 32). The correlation coefficient is 0.814, indicating a high positive correlation. EtCO_2_, end‐tidal carbon dioxide; PaCO_2_, partial pressure of arterial carbon dioxide; PvCO_2_, partial pressure of venous carbon dioxide

**FIGURE 3:**
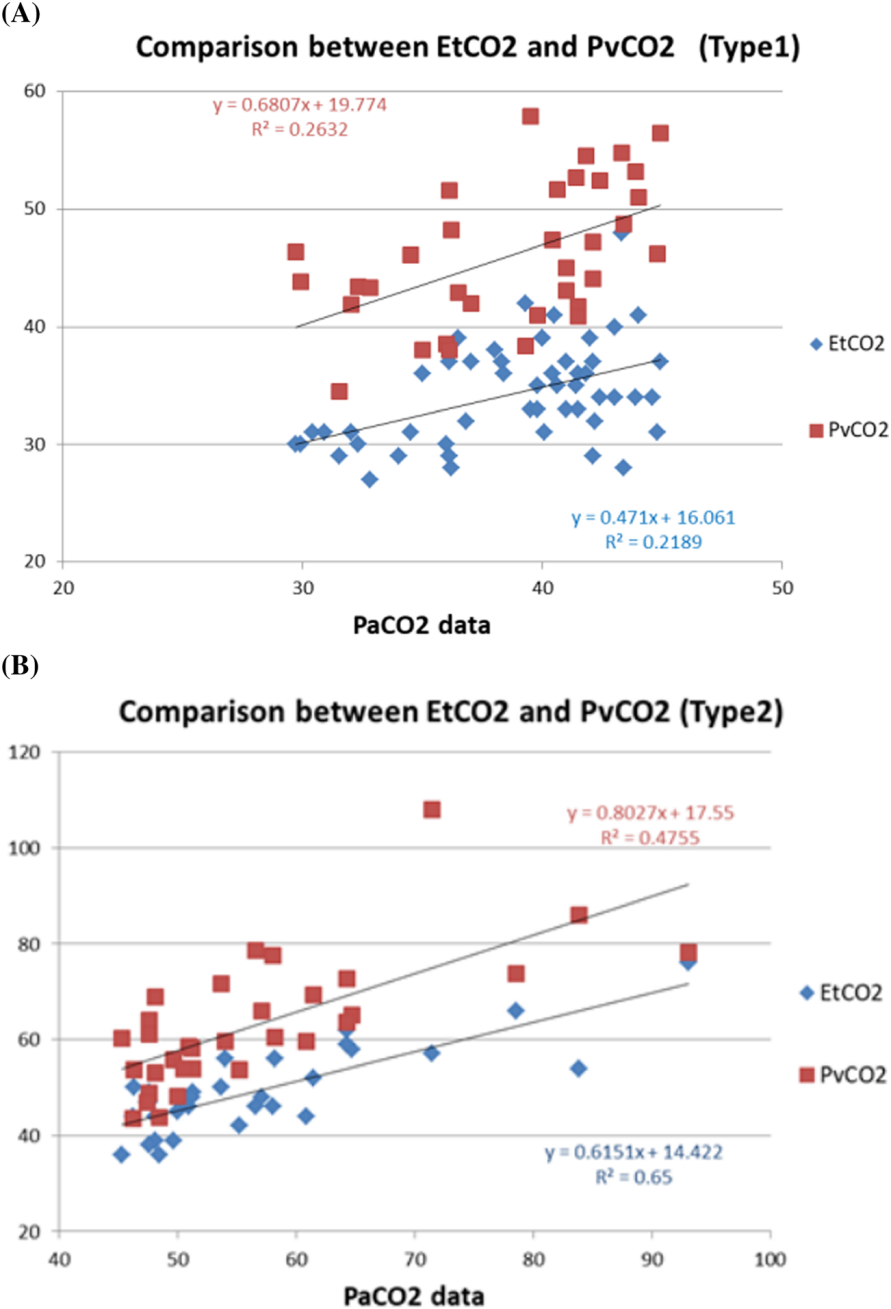
Separated Type 1 and Type 2 respiratory failure patients and comparison between EtCO_2_ and PvCO_2_. (A): A comparison between EtCO_2_ and PvCO_2_ in patients with Type 1 PaCO_2_ (EtCO_2_: *n* = 51, PvCO_2_: *n* = 34). The correlation coefficient of EtCO_2_ is 0.468 and that of PvCO_2_ is 0.513. Both data show a low positive correlation in Type 1 patients. (B) Correlation between EtCO_2_ and PvCO_2_ in Type 2 patients (EtCO_2_: *n* = 49, PvCO_2_: *n* = 32). The correlation coefficient of EtCO_2_ is 0.806, and the correlation coefficient of PvCO_2_ is 0.69. While 0.806 is considered a high positive correlation, 0.69 is interpreted as a moderate positive correlation. EtCO_2_, end‐tidal carbon dioxide; PaCO_2_, partial pressure of arterial carbon dioxide; PvCO_2_, partial pressure of venous carbon dioxide

No specific technical problems were encountered in these measurements. Figure 2A depicts the EtCO_2_ and PaCO_2_ in all patients (Type 1: *n* = 51, Type2: *n* = 49). The correlation coefficient was 0.883, and we observed a high positive correlation. (*r* = 0.88; *p* < 0.0001). Figure 2B depicts the relationship between PvCO_2_ and PaCO_2_ in all patients (Type 1: *n* = 34, Type 2: *n* = 32), with a correlation coefficient of 0.814, which demonstrated a high positive correlation (*r* = 0.81; *p* < 0.0001). Next, we separated the data into two categories, Type 1 and Type 2, and illustrated the correlations between them in the figures (Figure 3A,B). While type 1 patients (*n* = 51) displayed a moderate correlation (*r* = 0.47), type 2 patients (*n* = 49) demonstrated a high correlation (*r* = 0.81) between EtCO_2_ and PaCO_2_. In contrast, the correlation between PvCO_2_ and PaCO_2_ was moderate for both type 1 (*n* = 34) and type 2 (*n* = 32) (*r* = 0.51 and *r* = 0.69, respectively).

**FIGURE 4:**
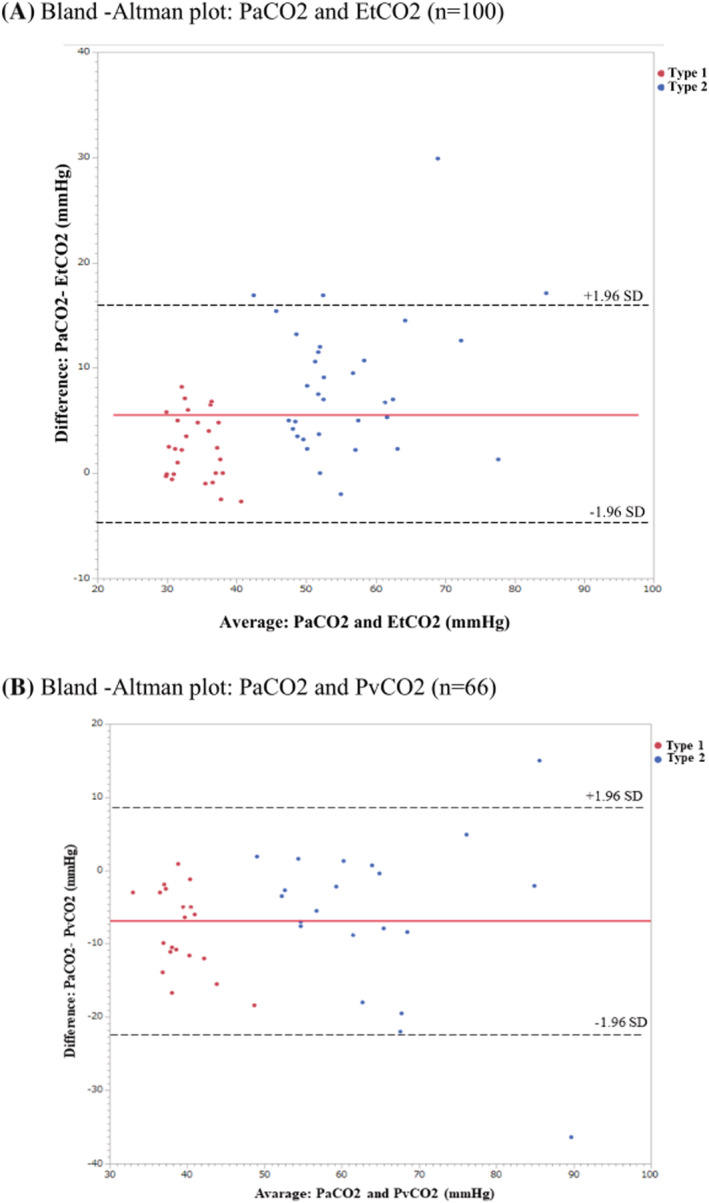
Bland–Altman analyses of respiratory gases in all subjects. (A) Comparison of PaCO_2_ and EtCO_2_ in all subjects, with a mean bias and limits of agreement. The solid line indicates 5.7 mmHg bias; dotted lines indicate the upper and lower limits of agreement (±1.96 SD). (B) Comparison of PaCO_2_ and PvCO_2_ in all subjects, with a mean bias and limits of agreement. The solid line indicates 6.8 mmHg bias; dotted lines indicate the upper and lower limits of agreement (±1.96 SD). EtCO_2_, end‐tidal carbon dioxide; PaCO_2_, partial pressure of arterial carbon dioxide; PvCO_2_, partial pressure of venous carbon dioxide

Figure 4 depicts agreements between PaCO_2_ and EtCO_2_ and between PaCO_2_ and PvCO_2_. The Bland–Altman analysis between PaCO_2_ and EtCO_2_ revealed a bias of 5.7 mmHg, with limits of agreement (bias ± 1.96 SD) ranging from −5.1 mmHg to 16.5 mmHg. However, it also revealed a bias of −6.8 mmHg between PaCO_2_ and PvCO_2_, with limits of agreement (bias ± 1.96 SD) ranging from −22.13 mmHg to 8.53 mmHg.

## 4 DISCUSSION

In our study, EtCO_2_ measured using the novel portable capnometer was significantly correlated with PaCO_2_ levels in patients with respiratory diseases. The correlation coefficient between EtCO_2_ and PaCO_2_ was 0.883, thus indicating a positive correlation (*r* = 0.88; *p* < 0.0001). Moreover, the correlation coefficient between PvCO_2_ and PaCO_2_ was 0.814 and also indicated a positive correlation (*r* = 0.81; *p* < 0.0001). The Bland–Altman analysis demonstrated good agreement and tolerance between the two variables. Furthermore, EtCO_2_ measured using the novel tool may be more useful for predicting hypercapnia conditions in which respiratory diseases are compared with measure PvCO_2_. However, the limits of agreement against both groups were wide. Therefore, care providers must pay attention to the characteristics and errors of these devices.

Conventionally, EtCO_2_ has been measured for the non‐invasive estimation of PaCO_2_. EtCO_2_ is commonly measured in the closed circuit in patients undergoing mechanical ventilation. Therefore, it has been most often used as a non‐invasive substitute for PaCO_2_ in anesthesia, anesthetic recovery, and intensive care.^8^ By improving the aforementioned measurement technologies, EtCO_2_ would more accurately predict PaCO_2_. Without significant changes in cardiac output or in the ventilation/perfusion ratio, PaCO_2_ can be estimated with clinically acceptable sensitivity and specificity than EtCO_2_.^9^ However, an accurate measurement of EtCO_2_ is challenging in patients who are not intubated. This can be attributed to the difficulty to collect pure respiratory gas.

Despite the usefulness of monitoring oxygen saturation (SpO_2_) for determining hypoxemia, measuring SpO_2_ alone is not sufficient to detect hyperventilation or hypoventilation. Particularly with the administration of supplemental oxygen, patients can display adequate arterial saturation during hypoventilation.^10^ However, pulse oximetry does not provide information on hypoventilation and hyperventilation. Therefore, it is clinically important to assess the status of CO_2_ retention in patients with respiratory failure. Nonetheless, no devices have been identified to easily monitor CO_2_ retention in general wards and outpatient clinics. Recently developed transportable capnometers can measure EtCO_2_ easily and non‐invasively in patients who are not intubated. Moreover, a recent study demonstrated that they could provide reliable EtCO_2_ values compared to PaCO_2_ in patients undergoing general anesthesia.^11^

CapnoEye® can measure EtCO_2_ easily and non‐invasively in non‐intubated patients. In this study, we examined its usefulness by considering the experience of use and the measured value. EtCO_2_ during general anesthesia in patients without lung disease is 2–5 mmHg lower than PaCO_2_.^12^ Our data also suggested that EtCO_2_ was 5.7 mmHg (standard deviation, 5.5) lower than PaCO_2_. We also confirmed a high correlation between EtCO_2_ and PaCO_2_ in patients with type 1 and 2 respiratory failure. In other words, the non‐invasive estimation of PaCO_2_ by measuring EtCO_2_ using CapnoEye® is clinically useful. The measurement of EtCO_2_ was not associated with any complications. In addition, the patient burden was extremely small. It is necessary to wait for few minutes for a 0‐point correction before the measurement. However, the measurement process is easy. The measurement only required disposable mouthpieces, and CapnoEye® was able to measure EtCO_2_ at a low price. However, its disadvantage was the inability to continuously monitor and record the parameter. While this measurement device is simple, based on a proprietary algorithm, and is easily measured and carried around, it does not enable offline storage of measurement data; therefore, it requires the user to take notes or pictures of the displayed data on the screen. It could not be used for patients with impaired consciousness or cognitive decline who were unable to respond to our instructions.

A previous study reported that arterial blood gas analysis during the acute exacerbation of chronic obstructive pulmonary disease could be substituted by venous blood gas analysis.^13^, ^14^ Venous blood is easier to collect for sample collection and more useful for multiple blood gas monitoring. However, the process of puncturing a blood vessel is still necessary. Therefore, a device that allows non‐invasive assessment would reduce the burden on both patients and clinicians. In our study, EtCO_2_ was correlated with PaCO_2_ higher than PvCO_2_, particularly in type 2 respiratory failure. The measurement of PvCO_2_ instead of PaCO_2_ would result in data discrepancies in patients with chronic respiratory failure, as in our patient cohort. The effects of cardiac output and tissue oxygen consumption may have caused differences in the venous and arterial CO_2_ levels in patients with chronic respiratory failure, compared with known reports. Therefore, our findings suggest that the predicate of arterial blood gas analysis likely preferred EtCO_2_ to PvCO_2_ in patients with chronic respiratory failure, predominantly type 2 respiratory failure. Type 2 monitoring is more important in actual clinical practice. This highlights the significance of performing non‐invasive monitoring in patients with a tendency for chronic CO_2_ retention.

## 5 LIMITATIONS

Our study had several limitations. First, the Bland–Altman analysis demonstrated that the limits of agreement against both groups were wide. Therefore, care providers must pay attention to the characteristics and errors of these devices. Second, this was a single‐center retrospective study comprising a small sample size. Third, the CapnoEye® measurement changes depending on the patient's breathing pattern. Therefore, it could only evaluate EtCO_2_ in patients who were alert and breathed spontaneously. Thus, it was unsuitable for patients who did not spontaneously breathe, were unable to respond to our instructions, or demonstrated poor spontaneous breathing, such as those with neuromuscular diseases. Fourth, some patients with type 2 respiratory failure underwent repeated measurements, which may be a source of bias. However, it is unknown whether EtCO_2_ measurements using this novel device in patients with refractory failure and spontaneous breathing can be considered an alternative to PaCO_2_ measurements that use arterial blood gas analysis in clinical settings.

## 6 CONCLUSION

Considering the improved accuracy of measuring instruments, EtCO_2_ measuring instruments have displayed a high correlation with PaCO_2_ and are clinically applied as a substitute for PaCO_2_. The novel portable capnometer can measure EtCO_2_ in a simple and non‐invasive way. EtCO_2_ measured using CapnoEye® demonstrated a positive, strong, and statistically significant correlation with PaCO_2_ levels in patients with respiratory diseases. Furthermore, EtCO_2_ measured using the novel tool may be more useful for predicting hypercapnia conditions in which respiratory diseases are compared to measure PvCO_2_.

We intend to conduct future studies to confirm the usefulness of these measurements, particularly for type 2 patients in primary care.

## CONFLICT OF INTEREST

None.

## ETHICS STATEMENT

This study was conducted in accordance with the tenets of the Declaration of Helsinki, and the protocol was approved by the Ethics Committee of Clinical Investigation for the National Center for Global Health and Medicine (approval number: NCGM‐G‐002135–00). Written informed consent was obtained from all patients.

## AUTHOR CONTRIBUTIONS

MS conceptualized and designed the study, drafted the initial manuscript, and reviewed and revised the manuscript. MS, SF, KS, and KT entered and collected data into the database. MS and SF contributed significantly to managing the data and MS to the statistical analysis. All authors revised the article critically for important intellectual content, gave their final approval of the version to be published, and agreed to be accountable for all aspects of the work.

## Data Availability

The data that support the findings of this study are available on request from the corresponding author. The data are not publicly available due to privacy or ethical restrictions.
